# T Lymphocyte Dynamics in Inflammatory Bowel Diseases: Role of the Microbiome

**DOI:** 10.1155/2015/504638

**Published:** 2015-10-25

**Authors:** C. B. Larmonier, K. W. Shehab, F. K. Ghishan, P. R. Kiela

**Affiliations:** ^1^Department of Pediatrics, Steele Children's Research Center, University of Arizona, Tucson, AZ 85724, USA; ^2^Department of Immunobiology, University of Arizona, Tucson, AZ 85724, USA

## Abstract

Humans have coevolved with a complex community of bacterial species also referred to as the microbiome, which reciprocally provides critical contributions to human metabolism and immune system development. Gut microbiome composition differs significantly between individuals depending on host genetics, diet, and environmental factors. A dysregulation of the symbiotic nature of the intestinal host-microbial relationship and an aberrant and persistent immune response are the fundamental processes involved in inflammatory bowel diseases (IBD). Considering the essential role of T cells in IBD and the contributing role of the microbiome in shaping the immune response during the pathogenesis of IBD, this review focuses on the complex relationship, interplay, and communication between the gut microbiome and T cells, including their differentiation into different subsets of effector or regulatory cells.

## 1. Introduction

Human inflammatory bowel disease (IBD) is a spontaneously relapsing, immunologically mediated disorder of the gastrointestinal tract, characterized by uncontrolled inflammation resulting from inappropriate and persistent activation of the mucosal immune system. Crohn's disease (CD) and ulcerative colitis (UC) represent the two most common forms of the condition, with associated significant morbidity and mortality [1]. Although both environmental factors and genetic predisposition have been implicated in the pathogenesis of IBD [2], the precise causes remain mostly undetermined.

Immune activation within the gut-associated lymphoid tissue (GALT) is essential to counteract potentially harmful pathogens. However, the control of such responses is necessary to avoid an inappropriate immune response against self- or harmless antigens. Disruption of any of the specific immune defense and regulatory mechanisms may lead to the development of chronic intestinal inflammation. Indeed, current evidence suggests that an inappropriate and persistent immune response against the intestinal microbiota plays a pivotal role in the pathogenesis of IBD [3].

The intestinal lamina propria is composed of complex immune cell populations which balance the requirement for physiologic immune tolerance to luminal antigens and the necessity to defend against pathogens. The hallmark of active IBD is an aberrant mucosal infiltration by innate immune cells (primarily neutrophils, macrophages, and dendritic cells) and adaptive immune cells (T and B cells). Effector CD4^+^ T cells (Th1, Th2, Th17, and T follicular helper Tfh) are critical in the defense against pathogens, whereas regulatory T cells (nTreg, iTreg; Tr1 and Th3) play a significant role in limiting the expansion and overactivity of CD4^+^ effector T cells. IBD seems to be due to either an excessive activation of effector T cells and/or alteration of T cell-mediated tolerance mechanisms, the latter through defects in the development of Treg or alteration in their immunosuppressive properties.

The intestinal microbiome has been the subject of intense focus in the last decade, as it is central in both the development of the intestinal immune system and the maintenance of immune tolerance. In particular, Toll-like receptors (TLRs), a family of pattern recognition receptors recognizing conserved commensal bacterial products (including lipopolysaccharide, DNA, and lipoteichoic acid), mediate protection from epithelial injury and play a crucial role in the maintenance of intestinal epithelial homeostasis in normal steady state conditions [4]. Additionally, it supplies essential nutrients, modulates energy metabolism, and participates in epithelial cell exfoliation. At least 200–300 different colonic species from 1,800 genera representing between 15,000 and 36,000 individual bacterial species constitute the total microbial load in the intestine [3]. In other terms, the lumen of the distal ileum and the colon contains 10^14^ bacteria, whereas the human body is composed of only 10^12^ cells. This extremely complex microbiota provides an important source of specific microorganisms, antigens, and ligands that can modulate the activation of the immune system. Indeed, microbial metabolites have a significant impact on disease progression and overall host health and play an important role in the maintenance of gut homeostasis [5].

Dysregulation of the symbiosis between the host intestine and its microbiota and an aberrant and persistent immune response are the fundamental processes involved in inflammatory bowel diseases. Taking into consideration the essential role of T cells in IBD and the contributing role of the microbiome in the pathogenesis of IBD, this review focuses on the complex relationship and interplay between the gut microbiome and T cell differentiation in the context of disrupted mucosal homeostasis in IBD.

## 2. T Lymphocytes in Immune Tolerance and Inflammation in IBD: General Concepts

Mucosal innate immune responses are a prerequisite for eliciting adaptive immune responses, as well as for eliciting the adaptive immune responses, which may become the major drivers of tissue injury and establishment of chronic inflammation. The innate components involve a complex network of nonprofessional and professional antigen presenting cells (APC), appropriate sensing of microbial-associated molecular patterns (PAMP) through specialized Toll-like receptors (TLRs) and the nucleotide oligomerization domain- (NOD-) like receptors (NLRs), and microbial control via the production of antimicrobial peptides and mucosal IgA. In recent years, innate lymphoid cells have also become recognized for their important role in mucosal immune homeostasis [6]. More detailed reviews on the innate elements contributing to tolerogenic and pathogenic adaptive immune response have been recently published elsewhere [7–11].

### 2.1. Role of T Cells in the Maintenance Immune Tolerance in the Gut Mucosa

Mucosa has developed a complex immune system that is capable of mounting an immune response against pathogens, while maintaining the required ignorance or active suppression against nonpathogenic antigens. Immune tolerance is the key mechanism by which intestinal homeostasis is maintained. It refers to the state of active hyporesponsiveness to dietary antigens, commensal enteric bacteria, and orally administrated substances, by a set of mechanisms which evolved to treat external harmless agents to which the immune system is systematically exposed, as an extension of “self.”

Several types of T cells exert active immunosuppressive effects in the gastrointestinal tract. “Innate-like” lymphocytes, represented primarily by the TCR*γδ* ^+^ T cells, provide signals that enhance barrier function and intercalate between intestinal epithelial cells on the basolateral side of epithelial tight junctions [12]. They respond to epithelial insults by secreting epidermal growth factor (EGF) to promote epithelial repair [13] and by producing proinflammatory cytokines and antimicrobial factors [14] which set the basal mucosal inflammatory tone and limit bacterial translocation.

Past research has shown anticolitogenic properties of CD8^+^CD28^−^ cells [15], CD8^+^CD122^+^ cells [16], and CD8^+^CD11c^+^ T cells [17]. More recently, Liu et al. [18] showed that,* in vitro*, TGF*β* can induce two distinct populations of CD8^+^Foxp3^−^ and CD8^+^Foxp3^+^ immunosuppressive T cells. They demonstrated that CD103 expression was obligatory for both populations to be immunosuppressive* in vitro* and to potently prevent CD4^+^ T cell-mediated colitis in* Rag2* ^−*/*−^ mice. This finding was consistent with an earlier report by Ho et al. [19], who described a CD8^+^CD103^+^ T cell population which produced TGF*β*, inhibited the proliferation of CD4^+^ *in vitro*, and attenuated adoptively transferred ileitis* in vivo*.

The most significant and most extensively studied immunosuppressive lymphocyte populations are TCR*αβ* CD4^+^ regulatory Treg cells, characterized by the expression of the Foxp3 transcription factor. Foxp3 mutations in humans lead to IPEX (immunodysregulation polyendocrinopathy enteropathy X-linked) syndrome, whereas mice lacking Foxp3 develop inflammation-mediated fatal multiorgan failure [20]. Other mutations leading to abnormalities in Treg numbers and function, such as in the WASP, CD25, or IL-10 genes, are also considered risk factors for IBD [21]. Two Foxp3^+^ Treg subsets are described: “natural” Treg of thymic origin (nTreg) and “induced” (iTreg), “adaptive,” or “peripheral” Treg cells. iTregs develop from naïve T cells in the periphery when activated by transient TCR stimulation or TCR stimulation in the presence of TGF*β* and IL-2. nTregs have the ability to recognize both self-antigens and foreign antigens and play an important role in the maintenance of self-tolerance and prevention of chronic immune stimulation in IBD [22]. After exiting the thymus, nTreg can migrate to the GI tract, where they prevent inappropriate immune responses. nTreg cells act synergistically with iTregs to prevent experimental colitis [23]. iTregs include two main subsets: FoxP3^+^ Th3 cells, secreting predominantly TGF*β*, and the Tr1 subset, characterized by lack of Foxp3 expression and high production of IL-10. Both subsets have been implicated in maintaining mucosal homeostasis and in protecting from experimental colitis [24–26]. More detailed reviews on the role of Tregs, their lineages, and respective functions in the intestinal mucosal homeostasis have been recently published [21, 27].

### 2.2. Types and Roles of Effector T Cells in the IBD-Associated Pathogenic Immune Responses

CD4^+^ T lymphocytes are believed to play a key role in the pathogenesis of human IBD, as evidenced by their influx into the inflamed mucosa, the effectiveness of depleting anti-CD4 antibody therapies [28], or the suppression of Crohn's disease symptoms observed in individuals with concomitant HIV infection [29]. The essential role of CD4^+^ T cells has been demonstrated in several animal models of experimental colitis, most notably in the adoptive naïve T cell transfer into a lymphopenic host [30]. Furthermore, this model of colitis is dependent upon the presence of bacteria, as it does not occur under germ-free conditions [31].

Traditionally, it was believed that CD was characterized predominantly by Th1 T cells secreting IFN*γ*, which were induced via the IL-12/IL-27-dependent differentiation pathway. In contrast, UC was thought to be mediated by Th2 T cells, associated with IL-4, IL-5, and IL-13 production. However, in recent years, the Th1/Th2 paradigm has been considerably revised. The role of Th17 cells in the adaptive immune response in IBD has been described. The Th17 subset differentiates from naïve T cells in the presence of IL-6 and TGF*β* and secretes IL-17 and IL-22. Primarily secreted by activated dendritic cells (DC) and macrophages, IL-23 is important in the maintenance of the Th17 cell lineage. Elevated IL-17 and IL-22 levels have been found in the gut mucosa of patients with UC and CD [32]. Importantly, a simplistic model of any one lineage driving the disease pathogenesis is difficult to prove. As an example, an enrichment of IFN*γ* ^+^IL-17^+^ coproducing CD4^+^ T cells with a proinflammatory phenotype, previously ascribed to CD pathogenesis, can also be found in actively inflamed mucosal lesions from UC patients [33]. Moreover, involvement of different Teff cells can change during the course of disease as exemplified by the SAMP1/YitFc mouse, a model of spontaneous CD-like ileitis. In these mice, ileitis initiation is mediated by a Th1 response with IFN*γ* and TNF*α* production, but the establishment of chronic inflammation is dependent upon a Th2 response, with secretion of IL-5 and IL-13 [34]. Another layer of complexity is added when T cell plasticity is considered, as exemplified by the Foxp3^+^ IL-17-producing CD4^+^ T cells enriched in active IBD [35, 36] or the aforementioned IFN*γ* ^+^IL-17^+^ CD4^+^ T cells. IL-21-producing follicular helper T cells (Tfh) have also been recently characterized in IBD patients, although IL-21 production can also be acquired by IFN*γ* ^+^ Th1 T cells [37].

The pathogenic role of autoreactive CD8^+^ T cells in IBD has been demonstrated in murine studies and implied by observations in IBD patients. Low avidity autoreactive CD8^+^ T cells can escape both central and peripheral tolerance and may trigger autoimmune reactions to a microbial mimic of self-antigen [38]. Funderburg et al. [39] recently documented elevated numbers of activated CD8^+^ T cells in IBD patients. Animal studies support the role of pathogenic MHC class I-restricted CD8^+^ T cells in response to self- or exogenous antigens in the intestinal mucosa [40–42].

## 3. Microbiome and T Cells

A metagenomic catalog of the human gut microbial gene catalog, using next generation sequencing approaches, was recently published [43, 44]. Ninety-nine percent of the microbial genes were bacterial. Approximately 160 species were identified, largely shared amongst individuals [43]. Gut commensal bacteria play critical roles in making nutrients accessible to the host, in the development of the immune system, and in the host's metabolism. In healthy individuals, the intestinal microbiome is dominated by four major bacterial phyla: Bacteroidetes, Firmicutes, Proteobacteria, and Actinobacteria. Reduced phylogenetic diversity has been described in IBD patients. Indeed, a relative decrease in Firmicutes (including Clostridia groups IV and XIVa) and an increase increase in Bacteroidetes, Proteobacteria, and Actinobacteria have been observed consistently among IBD patients [45–47]. The pathogenic role of commensal bacteria in IBD is implicated by the fact that most germ-free (GF) colitis-susceptible rodents have no intestinal inflammation or immune activation but rapidly develop pathogenic immune responses and disease after colonization with specific pathogen-free enteric bacteria [48]. Moreover, GF mice have relatively underdeveloped GALT [49]. Therefore, specific members of the gut microbiota were postulated to orientate host-specific T cell responses and modulate T cell differentiation.

### 3.1. Effect of the Microbiome on Effector T Cells

The importance of the microbiome relative to T cells is evidenced by the fact that the overall numbers of both CD4^+^ and CD8^+^ T cells are decreased in germ-free mice but are restored after recolonization [50] and that gut immune maturation is dependent upon colonization with host-specific microbiota [51]. However, upon infection with the intestinal parasite* Toxoplasma gondii*, CD4^+^ T cells respond against the pathogen as well as the translocating commensal bacteria, demonstrating the ability of the immune response to override tolerance to commensals [52]. The key role of the microbiota on T cell plasticity has been illustrated by the recent isolation of T cell clones specific for bacteria such as the Enterobacteriaceae,* Bacteroides*, and* Bifidobacterium* [53]. Functional relevance of those bacteria specific T cell clones was demonstrated by the transfer of clostridial flagellin-specific CD4^+^ effector T cells into* Toxoplasma*-infected mice, which are able to induce inflammation in the colon [54].

*Bacteroides fragilis*, a gram-negative anaerobic bacteria and its capsular polysaccharide A (PSA) have been described to modify the Th1/Th2 T cell balance in mice. This mechanism was shown to be dependent upon the PSA zwitterionic charge motif, which allows for its processing by dendritic cells. PSA signals through TLR2 and initiates the production of cytokines modulating Th1/Th2 balance (activation of NF-*κ*B, regulation of TNF*α*, IL-12 production). PSA function in the regulatory arm of the adaptive immune system is described in more detail below [55].

In the absence of commensal bacteria, germ-free or antibiotic-treated mice have significantly fewer Th17 cells than under normal conditions. It is well accepted that commensal bacteria prime Th17 differentiation [56]. In particular, adenosine 5′-triphosphate (ATP) derived from commensal bacteria has been shown to activate CD11c^low^CD70^high^ cells in the lamina propria, driving Th17 cells differentiation [57]. In addition, certain commensal bacteria can modulate the production of IL-17 by gut resident *γδ*T cells in the small intestine. Duan and colleagues have demonstrated that commensal bacteria participate in the expansion and maintenance of CD62L^−^ IL-1R^+^ *γδ*T cells and that IL-1 signaling is necessary for the secretion of IL-17 by *γδ*T cells, highlighting the essential role of endogenous flora [58]. In addition, some commensal bacteria can modulate the production of IL-17 by gut resident *γδ*T cells in the small intestine [58]. One microbial group collectively termed Segmented Filamentous Bacteria (SFB), a nonculturable group of Clostridia-related organisms, have been shown to play a role in the differentiation of the Th17 T cell lineage as well as IL-22-producing CD4^+^ T cells in the small intestine and the colon [59, 60]. The specific role of SFB in inducing Th17 responses remains unclear, although it is believed that SFB can stimulate the production of the early inflammatory marker serum amyloid A (SAA), which in turn stimulates a specific Th17 response* in vitro* [60]. Moreover, colonizing germ-free mice with SFB partially restores the *αβ*TCR IEL population, highlighting the importance of SFB on gut immune maturation [51]. SFB mediate protection against enteric infection by* Citrobacter rodentium* [59, 60]. Taken together, these findings suggest that SFB play a dual role in the maintenance of gut homeostasis. SFB are present in increased numbers in UC patients, suggesting their potential importance in human disease [61]. Lastly, thymic stromal lymphopoietin (TSLP), which is constitutively expressed in the intestine in the presence of intestinal bacteria, can play a role in the expansion of Th17 cells and also promotes Helios^−^Foxp3^+^ regulatory T cells [62].

GF animals harbor similar numbers of “innate-like” TCR*γδ* T cells compared to conventionally housed mice, yet under GF conditions these cells have a diminished ability to enhance mucosal repair [14]. TCR*γδ* T cells are able to control opportunistic translocation of commensal bacteria following intestinal epithelial injury and can be induced by commensal bacteria to produce antimicrobial peptides (Reg3*γ* by IEL and LPL TCR*γδ* T cells). The cumulative effect is a direct contribution to modulating the composition of the microbiota as well as containing invading enteric pathogens [14, 58].

Commensal bacteria also modulate the balance between regulatory and effector T cells via pattern recognition receptors, including TLRs. For instance,* Tlr9* ^−/−^ mice have an increased frequency of Treg and reduced IFN*γ*- and IL-17-producing Teff cells in the small intestine. This phenotype is reversed with administration of TLR9 ligands, indicating that commensal DNA or CpG have the ability to guide the mucosal responses to infection by a TLR9-dependent mechanism [63]. The role of TLR5 is also prominent, albeit complex. TLR5 stimulation promotes Teff cells while opposing Treg generation* in vitro*.* Tlr5* ^−/−^ mice develop spontaneous colitis, despite an increased number of Foxp3^+^ Tregs compared to WT littermates. TLR5-deficient mice have transiently increased levels of Proteobacteria, especially Enterobacteriaceae species (including* E. coli*), observed in close proximity to the gut epithelium. This has been attributed to a defect in innate sensing, which leads to unstable gut microbiota and low-grade inflammation, which may represent drivers for chronic colitis [64]. In the adaptive branch, dendritic cell stimulation by flagellin/TLR5 leads to an increase in IL-23, which further promotes the Th17 effector cell pathway to the detriment of Foxp3^+^ Treg [65, 66]. Moreover, certain flagellins have been postulated to be dominant antigens in the pathogenesis of CD. C3H/HeJBir mice, a substrain which develops spontaneous colitis, were used to identify a family of related novel flagellins, which activate TLR5, as a class of immunodominant antigens [67]. These flagellin clones were closely related to flagellins from* Butyrivibrio*,* Roseburia*,* Thermotoga*, and* Clostridium* within the* Clostridium* subphylum cluster XIVa. Antibodies to Cbir1, one of the commensal-derived flagellins, were also detected in* Mdr1* ^−/−^ and* Il-10* ^−/−^ mice and in CD patients, while Cbir1-specific CD4^+^ T cells induced severe colitis when adoptively transferred into naive SCID mice [67].

### 3.2. Effect of the Microbiome on Regulatory T Cells

Although regulatory T cells play a critical role in the maintenance of gut homeostasis, commensal bacteria may not be required for the generation of Tregs in the small intestine. Indeed, Treg frequency and immunosuppressive function are the same in the small intestine of germ-free mice compared to SPF mice [63, 68]. However, colonic Treg are significantly reduced in germ-free mice, suggesting a critical role of the colonic microbiota in Treg generation [69].

The Ikaros-family transcription factor Helios, a putative marker for thymic nTregs, has been used to study the impact of nTreg in the colon and the role of the microbiota in differentiation of naïve peripheral T cells into colonic Tregs. In GF mice, colonic Tregs are Helios^high^, in contrast to conventional mice, in which colonic Tregs are mostly Helios^low^. This suggested that colonic Tregs are converted from CD4^+^ T cells in the periphery [70]. In conventional mice, colonic Tregs are therefore primarily differentiated outside the thymus in the gut in response to foreign antigens. These findings were replicated using another marker for thymic-derived Treg, neuropilin 1 [70].

The ways by which bacterial-derived molecules coordinate peripherally generated Treg are illustrated in work done with Clostridia and* Bacteroides fragilis*, as both increase the frequency and stimulate the function of colonic Treg. Similar to what has been observed with effector T cells, colonic commensal luminal antigens can shape the intestinal Treg pool via the selection of specific TCRs and promote the efficient generation of peripheral Treg tolerant of the commensal microbiota [70].

One of the best examples of Treg modulation by the microbiome comes from studies on* Bacteroides fragilis*. Capsular lipopolysaccharide A (PSA) is primarily responsible for the immunostimulatory and immunoregulatory potential of* B. fragilis. *PSA has been shown to signal through the TLR2/MyD88 pathway in dendritic cells and also directly on CD4^+^ T cells.* B. fragilis* promotes IL-10-producing Foxp3^+^ Treg [71, 72]. As mentioned above, GF mice have a general defect in colonic CD4^+^ T cell development;* B. fragilis* is able to correct these deficiencies [71]. Treg induction by* B. fragilis* is driven by PSA and is dependent upon TLR2 signaling to induce IL-10 production (increased mRNA for IL-10, TGF*β*2, GITR, ICOS, CTLA4, and Ebi3; IL-27*β*), an effect that is restricted to the Foxp3^+^ Treg population. These observations constitute some of the seminal evidence describing the specific ways an individual constituent of the microbiota can control inflammatory responses.

More recently, the role of Clostridia, primarily clusters IV (*Clostridium leptum*) and XIVa (*Clostridium coccoides*) in intestinal homeostasis, has been investigated. These clusters were found to be responsible for the accumulation of CD4^+^CD25^+^Foxp3^+^ Treg (Helios^−^, CTLA4, and IL-10^high^) in the colonic lamina propria [73] and suggested that Clostridia are potent inducers of peripheral Treg differentiation and proliferation, presumably in the gut. Reconstitution of germ-free mice with a mix of 46 Clostridial strains induced the accumulation of a significant number of Tregs to levels similar to those of SPF mice in the cecum and the colon (but not in the small intestine). The specific effect of* Clostridium* species on Treg was further emphasized by the fact that colonization of germ-free mice by 16* Bacteroides* species, SFB, and 3* Lactobacillus* specieshad a minimal effect on colonic Treg accumulation [73]. In addition, early colonization of SPF mice with Clostridia generated a Clostridia-rich environment, which was protective from experimental colitis. Similarly, the colonization of GF mice with 17 Clostridia species from human fecal microbiota was also capable of Foxp3^+^ Treg induction and inhibition of colitis in TNBS and adoptive T cell transfer models [74]. Tregs from Clostridia-treated colitic mice were competent to inhibit OT-I TCR CD8^+^ T cell antigen specific proliferation* in vitro*. This inhibition was even greater with the addition of autoclaved cecal contents from Clostridia-treated mice. Clostridia-mediated Treg generation appears, therefore, to be antigen-specific [74].

The protective effect of Clostridia species may be mediated to a large extent by SCFA (short chain fatty acids; acetic acid, propionic acid, and butyric acid). SCFA are believed to support the expansion of the existing pool of colonic Tregs, and butyrate alone has been demonstrated to induce Treg differentiation both* in vivo* and* in vitro*, enhancing histone acetylation in the promoter and conserved regions of the* Foxp3* locus [75, 76]. In another study, Singh et al. [77] showed that, in colonic antigen presenting cells, signaling through butyrate and the niacin receptor Gpr109a, coded by* Niacr1* gene, permits differentiation of Treg cells and IL-10-producing T cells. Gpr109a was required for the expression of IL-18, and* Niacr1* ^−*/*−^ mice showed enhanced susceptibility to colitis and colon cancer. Moreover, depletion of gut microbiota or dietary fiber increased the risk for colitis and cancer, which was suppressed by niacin in a Gpr109a-dependent manner [77].

The probiotic VSL#3 (comprised of* Bifidobacterium breve*,* Bifidobacterium longum* subsp.* infantis*,* Lactobacillus acidophilus*,* Lactobacillus plantarum*,* Lactobacillus paracasei*,* Lactobacillus bulgaricus*, and* Streptococcus thermophilus*) reduced the severity of experimental colitis, which correlated with diminished effector T cell cytokine levels [78, 79] and with increased number of TGF*β*- and IL-10-producing Tregs [80]. In addition,* Bifidobacterium longum* subsp.* infantis* has been shown to prevent* Salmonella typhimurium*-driven inflammation by increasing Treg numbers [81].

Recently, another gut commensal bacteria,* Faecalibacterium prausnitzii*, which typically is found in reduced abundance in IBD patients [82], was found to induce CD4^+^CD25^+^Foxp3^+^ Treg [83].

## 4. T Cells Can Also Shape the Gut Microbiome

Although less well-studied, there is emerging evidence which demonstrates the reciprocal relationship between T cells and their ability to shape the composition of the gut microbiota. Indeed, defects in competent adaptive immunity can modulate the response to the microbiota and has been linked to substantial changes in microbial composition. In a recent study, Zhang et al. [84] studied the microbiome composition in* Rag1* deficient mice and WT mice. This report demonstrated that the phylogenetic composition of the microbiota was different in* Rag1* ^−/−^ before and after weaning. In particular,* Akkermansia muciniphila* was enriched in immunodeficient mice. The lack of B and T cells also correlated with a decrease in Lactobacillales and Enterobacteriales in the pre-weaning stage and increased Verrucomicrobiales in the pre- and postweaning periods. Furthermore,* Rag1* ^−/−^ mice showed a significant increase in microbial diversity with age compared to WT, pointing out the role of adaptive immune system pressure in modulating gut microbiota composition [84]. SFB have also been shown to be more abundant in* Rag2* ^−/−^ mice [85]. In a recent study, Brugman and colleagues [86] analyzed the role of the adaptive immune system on* Rag1* ^−/−^ deficient zebrafish and showed that* Vibrio* species abundance increased. They also confirmed that adoptive T cell transfer in* Rag1* deficient zebrafish suppressed the overgrowth of* Vibrio*. The study was completed by an elegant* ex vivo* experiment where exposure of intestinal T lymphocytes to* Rag1* ^−/−^ derived microbiota increased IFN*γ* expression.

TRUC mice (*Tbet* ^−/−^  ×* Rag2* ^−/−^) display a defect in competent adaptive immune responses and develop spontaneous colitis, which can be controlled by Treg or antibiotic treatment.* Klebsiella pneumoniae* and* Proteus mirabilis* were found to be present in abundance in TRUC mice, linking a defect in the response to the microbiota to changes in the microbial composition [87]. Kawamoto et al. [88] described that Tregs are necessary for the maintenance of microbial diversity, including a promoting effect on the abundance of nonpathogenic Clostridia. These and other studies described in this review point to the elaborate and interdependent relationship between T cells, Treg in particular, and the gut microbiome.

## 5. Conclusions

**Figure 1:**
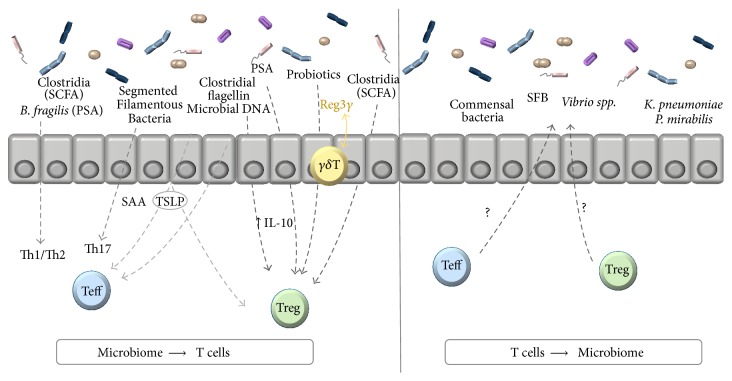
Schematic depiction of the major points emphasized in the review.

The studies outlined in this review provide strong evidence that the gut microbiota and CD4^+^ T cell relationship is dynamic and interdependent, with each affecting the composition/function of the other (Figure 1). The microbial composition of the host at different sites (small intestine or colon) plays a major role in the balance of Treg/Teff in the gut, whether this is driven by the production of microbial metabolites (e.g., SCFA), by colonization by SFB or other organisms, or through innate signaling via TLRs (mostly TLR5 and TLR9). In this review, we focused on the role of the microbiota on effector and regulatory T cells. The role of antecedent stimulation of the innate immune system as a prerequisite for T cell activation and dysregulation in colitis (the so-called “two-hit” model) is discussed elsewhere [89] and was beyond the scope of this review.

As our knowledge of the nuanced relationship between the microbiota and the adaptive immune system develops, it is tempting to speculate about the therapeutic potential of manipulating the microbiota to influence T cell responses and* vice versa* in IBD. Therapeutic manipulation of the microbiome for various other indications, including fecal transplantation for* Clostridium difficile* colitis and administration of probiotics, has been the subject of significant recent attention but has not yet been translated to the management of IBD [90]. Considering the mutualistic coevolution of the gut microbiome and host immune maturation, investigating this complex relationship has the potential to positively impact patients with IBD. Additional studies that translate the findings described in this review into clinical therapeutics are needed and highly anticipated.
